# Activation of T Lymphocytes with Anti-PDL1-BiTE in the Presence of Adipose-Derived Mesenchymal Stem Cells (ASCs)

**DOI:** 10.1155/2023/7692726

**Published:** 2023-06-07

**Authors:** Leila Moeinzadeh, Amin Ramezani, Fereshteh Mehdipour, Mahsa Yazdanpanah-Samani, Mahboobeh Razmkhah

**Affiliations:** ^1^Department of Tissue Engineering and Applied Cell Sciences, School of Advanced Medical Sciences and Technologies, Shiraz University of Medical Sciences, Shiraz, Iran; ^2^Shiraz Institute for Cancer Research, School of Medicine, Shiraz University of Medical Sciences, Shiraz, Iran; ^3^Department of Medical Biotechnology, School of Advanced Medical Sciences and Technologies, Shiraz University of Medical Sciences, Shiraz, Iran

## Abstract

**Background:**

Due to their ability to recruit immune cells to kill tumor cells directly, bispecific T cell engager antibodies (BiTE) hold great potential in T cell redirecting therapies. BiTE is able to activate T cells through CD3 and target them to tumor-expressed antigens. However, there are many components in the tumor microenvironment (TME) such as mesenchymal stem cells (MSCs) that may interfere with BiTE function. Herein, we designed an anti-PDL1-BiTE that targets programmed death ligand 1 (PDL1) and CD3 and investigated its effect on PDL1pos cancer cells in the presence or absence of adipose-derived MSCs (ASCs).

**Method:**

Our anti-PDL1-BiTE comprises of VL and VH chains of anti-CD3 monoclonal antibody (mAb) linked to the VL and VH chains of anti-PDL1 mAb, which simultaneously bind to the CD3*ε* subunit on T cells and PDL1 on tumor cells. Flow cytometry was employed to assess the strength of binding of anti-PDL1-BiTE to tumor cells and T cells. Cytotoxicity, proliferation, and activation of peripheral blood lymphocyte (PBLs) were evaluated by CFSE assay and flow cytometry after using anti-PDL1-BiTE in the presence or absence of ASCs and their conditioned media (C.M.).

**Results:**

Anti-PDL1-BiTE had the ability to induce selective lysis of PDL1pos U251-MG cancer cells while PDL1neg cells were not affected. Also, anti-PDL1-BiTE significantly stimulated peripheral blood lymphocyte (PBL) proliferation and CD69 expression. ASCs/C.M. did not show a significant effect on the biological activity of anti-PDL1-BiTE.

**Conclusion:**

Overall, anti-PDL1-BiTE selectively depletes PDL1pos cells and represents a new immunotherapeutic approach. It would increase the accumulation of T cells and can improve the prognosis of PDL1pos cancers in spite of the immunomodulatory effects of ASCs and C.M.

## 1. Introduction

The discovery of efficient anticancer approaches has been impeded by the complexity of malignancies [1, 2]. Certain conventional cancer therapies, such as surgery, chemotherapy, and radiation, are limited due to their associated side effects, off-target effects, and drug resistance [3, 4]. Furthermore, traditional therapies cannot entirely eradicate metastatic tumor cells, and relapse is likely to occur. As a result, researchers are attempting to develop new and effective therapies with low or no toxicity towards normal cells [5]. Now, cancer immunotherapy (CI) is rapidly advancing [6] and is mostly directed on the immune system or the tumor microenvironment (TME) rather than tumor cells themselves [7–9]. In the course of cancer treatment, TME is also a critical issue. TME includes extracellular matrix (ECM), stromal cells such as fibroblasts and mesenchymal stem cells (MSCs), vascular and lymphatic networks, and cells of the immune system [10–12]. In fact TME is a dynamic heterocellular environment [13]; that is, its cellular component is critical in the development and proliferation of cancer progenitor cells, such as tumor-initiating cells (TICs) and cancer stem cells (CSCs) [14, 15]. Because of their ability to change TME architecture, a wide variety of stem cells known as mesenchymal stem cells have picked the interest of cancer researchers [16–18]. There is a dual relationship between MSCs and tumor cells directly (cell-cell contact) and indirectly (by several soluble factors, exosomes, and other extracellular vesicles (EVs)) [19], so that primary and metastatic tumors actively recruit MSCs from the bone marrow and adipose tissue and develop either tumor inhibition or tumor promotion based on the tumor type [15]. MSCs' paracrine immunoregulatory capabilities through chemokines, cytokines, and growth factors such as stromal cell-derived factor 1 (SDF-1), IL-6, tumor growth factor- (TGF-) *β*, tumor necrosis factor-inducible gene 6 protein (TSG-6), and metabolites [20] as well as their cell-cell contact impact tumor growth and treatment [21].

Active T lymphocytes recognizing tumors are required for a successful immune response to cancer [22]; however, they cannot be recruited by conventional antibodies [23]. Bispecific T cell engagers (BiTEs) are genetically engineered recombinant proteins [24, 25] and a member of the family of dual antibodies. They have several different binding sites that simultaneously bind to the T cell receptor (TCR) CD3*ε* subunit on T cells and surface molecules on targeted tumor cells and recruit T cells to mediate cytotoxicity against tumor cells and creating the immunological synapse [22, 26, 27]. T cell activation occurs only in the presence of target cells, independent of TCR specificity, costimulation, or peptide antigen presentation. T cells release cytotoxic chemicals such as perforin and granzymes [28] after synapse formation, which trigger target cell apoptosis by caspases [27]. In the meanwhile, numerous studies in mice and patients with cancer have demonstrated that tumor cells contribute to the immune suppression [29] by recruiting or induction of the distinct cell populations, such as myeloid-derived suppressor cells (MDSCs) and T regulatory cells (Tregs) [30]. Moreover, tumor cells induce anergy or apoptosis in T cells via different immune checkpoint molecules (cytotoxic T lymphocyte-associated protein 4 (CTLA-4) and programmed death 1 (PD1)) [31]. Therefore, clinical trials using immune checkpoint inhibitors like PD1 or programmed death ligand 1 (PDL1) inhibitors significantly increased the survival of the patients and converted the “cold” tumors to “hot” ones [32].

In this study, we investigated whether anti-PDL1-BiTE can affect T cell biological activities like cytotoxicity, proliferation, and activation and also examined the effects of MSCs or their condition media on anti-PDL1-BiTE performance. Based on our results, anti-PDL1-BiTE had the ability to induce selective lysis of PDL1pos U251-MG cancer cells while PDL1neg cells were not affected. Additionally, anti-PDL1-BiTE significantly stimulated peripheral blood lymphocyte (PBL) proliferation and CD69 expression. ASCs/C.M. did not show a significant effect on the biological activity of anti-PDL1-BiTE.

## 2. Subjects and Methods

### 2.1. Subjects and Cells

Adipose-derived mesenchymal stem cells (ASCs) were extracted from adipose tissues which were provided from three patients with liposuction surgery with the age range of 20 to 30 years. Peripheral blood mononuclear cells (PBMCs) were obtained from five healthy donors with no record of cancer or autoimmunity. Cell lines, including human brain tumor cell line U-251 MG (PDL1-positive cell) and human embryonic kidney tumor cell line, HEK293, and JURKAT cells (PDL1-negative cells), were obtained from Stem Cell Technology Research Center (Bonyakhteh) and Pasteur Institute, Tehran, Iran. The R&D Laboratory (Shiraz Institute for Cancer Research, Shiraz University of Medical Sciences, Shiraz, Iran) generously provided the CHO-K1 cell line. This study was approved by the Ethics Committee of Shiraz University of Medical Sciences (ethics reference number IR.SUMS.REC.1399.306).

### 2.2. Antibodies

Antibodies for ASC characterization included mouse anti-human CD14 (clone: M5E2, cat. no. 555397, FITC), CD44 (clone: G44-26, cat. no. 560977, FITC), CD34 (clone: 581, cat. no. 555821, FITC), and CD166 (clone: 3A6, cat. no. 559263, PE) all from BD Biosciences, USA. Antibody to human PDL1 (CD274, B7-H1, clone: 29E.2A3 mAb, cat. no. 329701, concentration: 0.5 mg/mL, PE) was from BioLegend, USA. Fluorescent-conjugated antibodies used for analyzing PBLs were CD4 (clone: RPA-T4, cat. no. 300530, Percp Cy5.5, BioLegend), CD25 (clone: M-A251, cat. no. 555431, FITC, BD), and CD69 (clone: FN50, cat. no. 310910, APC, BioLegend,). Other antibodies were monoclonal anti-polyhistidin-peroxidase antibody (cat. no. A7058, HRP-conjugated, Sigma, USA), mouse monoclonal MMP8 (clone 115-13D2, cat. no. ab77964, Abcam), HRP-conjugated goat anti-human IgG (cat. no. A0170, Sigma, USA) for ELISA test, and goat anti-mouse IgG (cat. no. sc-3738, PE, Santa Cruz Biotechnology).

### 2.3. Cell Lines and ASCs' Culture

Cell lines were cultured in RPMI1640 medium (Biosera, UK) supplemented with 10% fetal bovine serum (FBS, Gibco, USA) and streptomycin (100 mg/mL) and penicillin (100 U/mL) (Biosera, UK) at 37°C in a humidified atmosphere with 5% CO_2_. CHO-K1 cells were cultured in CD OptiCHO Medium (Life Technologies) supplemented with 8 mM Glutamax (Gibco, Japan). ASCs were isolated from adipose tissues according to the previously established method [33, 34]. Briefly, adipose tissues were washed with sterile saline buffer, grinded, then treated with 0.2% type I collagenase (Gibco, USA) for enzymatic digestion, and incubated at 37°C. Subsequently, the suspension was centrifuged. The stromal vascular fraction (SVF) was separated using Ficoll (Biosera, UK) and cultured in DMEM (Biosera, UK) supplemented with 10% FBS and 1% penicillin/streptomycin. Following 48 hours culture, nonadherent cells and debris were washed out and new medium was added. Adherent and spindle-shaped ASCs were subcultured, and cells of the third passage was used for the following experiments.

### 2.4. ASC Characterization

ASCs were stained with fluorochrome-conjugated anti-human antibodies against cell surface antigens including CD14, CD44, CD34 and CD166, according to the previously established method [35]. The negative controls used for cell staining were FITC- and PE-labeled mouse IgG1. ASCs were stained with the aforementioned antibodies for 30 minutes at 4°C in the dark and were washed twice by staining buffer (PBS+2%FBS). The presence of surface markers was then evaluated using FACS Calibur flow cytometer (BD Biosciences, USA). The flow cytometry data were analyzed using FlowJo software (Version 10.1, Ashland, OR, USA).

### 2.5. Isolation of Human Peripheral Blood Lymphocyte (PBLs)

Peripheral blood mononuclear cells (PBMCs) used in this study were isolated from 5 healthy donors, using Ficoll-Hypaque (Biosera, UK) density gradient centrifugation and then were cultured in complete RPMI1640 medium at 37°C in a humidified atmosphere containing 5% CO2. To remove monocytes, PBMCs were incubated in RPMI1640 medium supplemented with 10% FBS and 1% penicillin/streptomycin at 37°C for 45 minutes. After incubation, the suspending cells were harvested as PBLs.

### 2.6. Harvesting of ASC-Conditioned Medium

To prepare ASC concentrated conditioned medium, ASCs (5 × 10^3^ cells/cm^2^) were cultured in DMEM supplemented with 10% FBS. Then, the media was changed at day 3 post seeding. When cells were approximately 80% confluent, they were washed twice with Ca- and Mg-free PBS; after that, the medium was replaced with serum-free DMEM. After 72 hours, the supernatant was collected and centrifuged to remove cellular debris at 1000 rpm for 15 minutes and used as ASC-C.M.

### 2.7. Construct Design and Generation of the Recombinant Protein

Initially, the sequences encoding the light and heavy chains (VL and VH regions) of avelumab, an anti-human PDL1 monoclonal antibody [36], and the VH and VL regions of the OKT3 anti-human CD3 monoclonal antibody [37] were extracted from several databases, such as DrugBank, PDB, and NCBI. The final construct was designed using these sequences along with appropriate linkers, secretory, and kozak sequences and a 6-residue histidine tag at the 3′ end. These fragments were sequence-optimized by GenScript's Multiparameter Gene Optimization algorithm, OptimumGeneTM (NJ, USA) and were synthetized by Biomatik (Ontario, Canada). The fragment was inserted at the AvrII/Bstz17I cloning site in the pCHO1.0 expression vector and transformed into DH5*α* cells. The anti-PDL1-BiTE construct was transfected into CHO-K1 cells by Gene Pulser Xcell electroporation system (Bio-Rad, CA, USA) (950 *μ*F, 300 V). Then, for selecting stable transfected cell pools based on increasing levels of puromycin (Gibco, Japan) and MTX (Sigma, USA), a two-phase selection strategy was used. Briefly, in selection phase 1, puromycin to a final concentration of 10 *μ*g/mL and MTX to 100 nM were used as selection reagents. Flasks were incubated at 37°C with 5% CO_2_, and the media were exchanged every 4 days until the confluency reached more than 90%. In selection phase 2, each recovered cell pool from selection phase 1 was subjected to puromycin to a final concentration of 30 *μ*g/mL and MTX to 500 nM. To obtain anti-PDL1-BiTE for experiments, transfectants were seeded in serum-free OptiCHO medium supplemented with 8 mM Glutamax (Gibco, Japan) and cultured for 8 days at 30°C. The HIS-tagged anti-PDL1-BiTE secreted by stably transfected cell pools. The supernatant was then collected, centrifuged to remove cellular debris, and stored at 4°C until use.

### 2.8. Determination of PDL1 Expression Level

The PDL1expression level on tumor cells was determined by flow cytometry. One day before the experiment, 10^4^ U-251 MG and HEK293 cells were plated in T25 flask. PDL1expression on the cell surface was detected by staining cells with PE-conjugated anti-human CD274 (B7-H1, PD-L1) and analyzed using a flow cytometer.

### 2.9. Physicochemical Properties of Anti-PDL1-BiTE Recombinant Protein

#### 2.9.1. Western Blot Assay

To analyze the purity of anti-PDL1-BiTE, native polyacrylamide gel electrophoresis (Native-PAGE) was used, which was visualized with R250 Coomassie blue (Bio-Rad, UK). The first method was performed as discussed by Ramezani et al. [38]. In brief, mock (as a negative control) and 60 ng/mL of the anti-PDL1-BiTE were loaded on a 12.5% Native-PAGE gel. Separated proteins were transferred to polyvinylidene difluoride (PVDF) membranes (GE Healthcare) using the Trans-Blot® Turbo™ Blotting System (Bio-Rad, UK) at 20 V for 60 minutes. After transferring to PVDF membrane and blocking with PBS containing 0.15% Tween 20 and 5% nonfat skim milk (Sigma, USA), the membrane was washed with PBS-Tween and incubated with HRP-conjugated anti-6×his antibody (1 : 2000 diluted). Signals were detected with enhanced chemiluminescence (ECL) substrate (Bio-Rad, USA), and the bands were visualized with Bio-Rad Chemidoc device (Bio-Rad, USA).

#### 2.9.2. Determining the Anti-PDL1-BiTE Concentration Using Enzyme-Linked Immunosorbent Assay (ELISA)

The supernatant of an 8-day culture of stably transfected CHO-K1 cells was collected for anti-PDL1-BiTE concentration analysis using direct ELISA. Briefly, a 96-well ELISA plate (PolySorp, NUNC™, Denmark) was coated with 50 *μ*L of serially diluted cell supernatant samples and incubated overnight at 4°C. A serial concentration of mouse monoclonal MMP8 antibody (1 to 1 × 10^7^ ng/l) was used to draw the standard curve. Then, 50 *μ*L HRP-conjugated goat anti-human IgG (1 : 20000) was added and incubated for 1 hour. Then, the plate was washed, and 50 *μ*L of tetramethyl benzidine (TMB) substrate (Invitrogen) was added. The reaction was stopped by the addition of 50 *μ*L of 0.25 M sulfuric acid (Merck, Germany) after 20 minutes. The plate was read on a standard absorbance microplate reader (Biochrom, UK) at 450 and 620 nm.

#### 2.9.3. Binding Assay

The binding ability of anti-PDL1-BiTE to PDL1-positive cells (U251-MG), CD3-positive cells (JURKAT), and PDL1- and CD3-negative cells (HEK293) was assessed. 1 × 10^6^ cells per sample were collected by centrifugation at 1000 rpm for 5 minutes and then washed with 1x PBS containing 0.2% FBS (staining buffer) in separate tubes. The cell pellet was resuspended in 100 *μ*L of ice-cold staining buffer and then incubated with 4 *μ*g/mL anti-PDLI-BiTE as the primary antibody on ice for 1 hour. After washing, the cells were incubated with PE-goat anti-mouse IgG (Santa Cruz Biotechnology) (1 : 1000 diluted) for another 1 hour on ice in the dark. Cells were then washed and resuspended in 300 *μ*L of PBS buffer. Cells were acquired on the FACS Calibur flow cytometer (BD), and data were analyzed using FlowJo.

### 2.10. Biological Activity of Recombinant Protein Anti-PDL1-BiTE

#### 2.10.1. Cytotoxicity Assay

Cytotoxicity assay was performed to determine the ability of PBL cells to eradicate PDL1pos tumor cells in the presence or absence of anti-PDL1-BiTE, ASCs, and C.M. PBLs, ASCs, and C.M. were used as effectors. Target cells (PDL1pos/neg tumor cells) were labeled using a Cell Trace™ CFSE Cell Proliferation Kit (C34554, Invitrogen, Thermo Fisher Scientific, Waltham, MA, USA) according to the manufacturer's instructions. One day before coculture, ASCs and CFSE-labeled tumor cells were seeded at a ratio of 1 : 5 (ASCs : tumor cells) in complete RPMI1640 medium and incubated at 37°C for 16 hours, in 24 well plate. Then, ASCs and CFSE-labeled tumor cells were directly cocultured with PBLs at the ratios of 1 : 100 (ASCs : PBLs) and 1 : 20 (tumor cells : PBLs). Anti-PDL1-BiTE (1.5 *μ*g/mL) and C.M. (400 *μ*L) were then added in a total volume of 2000 *μ*L in 24-well flat bottom plates and incubated at 37°C for 72 hours. The control groups contained tumor cells alone and tumor cells cocultured with PBLs/ACS/C.M. without anti-PDL1-BiTE. At the end of the incubation period, CFSE-labeled adherent tumor cells were harvested with 1% trypsin–EDTA, stained with 7AAD (BD Pharmingen™ cat. no. 5168981E), and analyzed for cytotoxicity using flow cytometry (BD Biosciences). The percentage of specific lysis was calculated as follows: %dead tumor cells = [tumor cells (7 − AAD+) − tumor cells (7 − AAD−)] × 100%.

#### 2.10.2. Induction of PBL Proliferation

Proliferation of T cells was detected using the Cell Trace CFSE Cell (Thermo Fisher Scientific) Proliferation Kit. Briefly, one day before coculturing, ASCs and the PDL1pos tumor cell line (U251-MG) were seeded at the ratio of 1 : 5 (ASCs : tumor cells) in complete RPMI1640 medium and incubated at 37°C for 16 hours in a 96 well plate. On the day of coculture, PBLs were stained with 5 *μ*M of CFSE for 15 minutes at 37°C. After washing, 1 × 10^5^ CFSE-labeled cells were directly cocultured with ASCs and tumor cells at the ratio of 1 : 100 (ASCs : PBLs) and 1 : 20 (tumor cells : PBLs) in a total volume of 250 *μ*L in 96-well flat bottom plates. Then, anti-PDL1-BiTE (1.5 *μ*g/mL) and C.M. (50 *μ*L) were added in complete RPMI1640 medium and incubated at 37°C for 72 and 96 hours. The control groups contained PBLs alone and PBLs cocultured with tumor cells/ASC/C.M. without anti-PDL1-BiTE. At the end of the incubation period, PBLs were harvested from the coculture and were analyzed for proliferation, using flow cytometry (BD Biosciences).

#### 2.10.3. Induction of PBL Activation

One day before coculturing, ASCs and PDL1pos tumor cells were seeded at a the ratio of 1 : 5 (ASCs : tumor cells) in complete RPMI1640 medium and incubated at 37°C for 16 hours in 24 well plate. Then, they were directly cocultured with PBLs at the ratios of 1 : 100 (ASCs : PBLs) and 1 : 20 (tumor cells : PBLs). Anti-PDL1-BiTE (1.5 *μ*g/mL) and C.M. (400 *μ*L) were then added in a total volume of 2000 *μ*L in 24-well flat bottom plates and incubated at 37°C for 48 hours. The control groups were PBLs alone and PBLs cocultured with tumor cells/ASC/C.M. without anti-PDL1-BiTE. At the end of incubation period, PBLs were harvested and washed with staining buffer and centrifuged at 4°C for 5 minutes before staining with labeled antibodies. PBLs were incubated with fluorochrome-conjugated antibodies to CD4, CD25, and CD69 at 4°C for 30 minutes in the dark. Finally, the stained cells were acquired and analyzed using a four-color FACS Calibur flow cytometer (BD Biosciences).

### 2.11. Statistical Analysis

Statistical analysis was performed using GraphPad Prism 9. Quantitative data are shown as the mean ± standard of deviation (SD). A one-way analysis of variance (ANOVA) was applied, followed by Tuckey's multiple comparison test and the evaluation of differences. *P* < 0.05 was considered statistically significant. Each test was done on 5 individuals and repeated once for each condition.

## 3. Results

### 3.1. Isolation, Culture, and Characterization of ASCs

**Figure 1:**
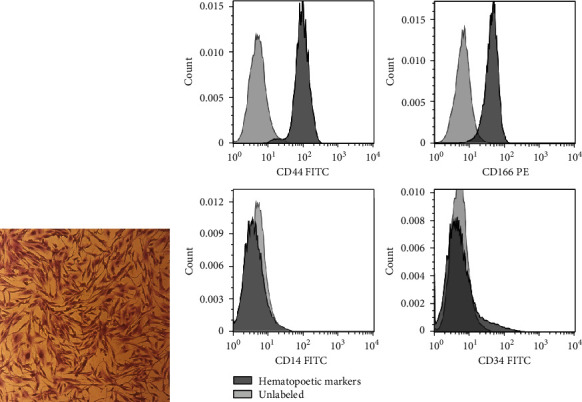
Morphological and flow cytometry analysis of adipose-derived mesenchymal stem cells (ASCs). (a) ASCs were spindle-shaped in culture. (b) More than 95% of ASCs were positive for CD44 and CD166, while they did not express hematopoietic markers including CD14 and CD34. CD markers were selected based on those proposed by International Society for Cellular Therapy (ISCT) and previous studies. The black color indicates hematopoietic markers, and the gray color indicates unlabeled.

ASCs were observed as an adherent cell population with a spindle-shaped appearance (Figure 1(a)). The analysis of the stem cell-specific markers revealed that the majority of ASCs were positive for CD44 and CD166, while they were negative for the expression of hematopoietic cell-specific markers such as CD14 and CD34 (Figure 1(b)).

### 3.2. Identification of Recombinant Anti-PDL1-BiTE

**Figure 2:**
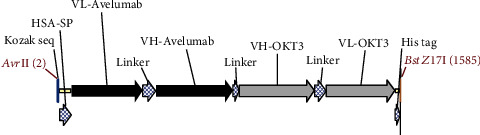
Schematic representation of anti-PDL1-BiTE construct. Each single-chain variable fragment (scFv) was composed of VH and VL domains of avelumab and OKT3, which were linked by a residual peptide linker. Kozak sequence and human serum albumin (HSA) located in 5th and His tag in 3rd.

**Figure 3:**
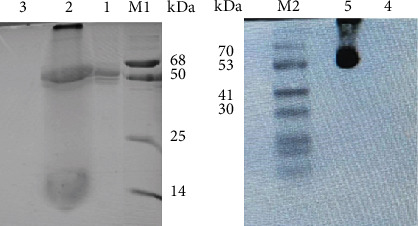
Physicochemical analysis of produced anti-PDL1-BiTE. (a) Native-PAGE. (b) Western blot. The supernatant from transfected CHO-K1 cells contained a specific ~55 kDa protein. The migration distances of the molecular mass markers are indicated in kilodaltons (kDa). M1: house laboratory ladder; M2: Sinaclon prestained protein ladder, 1 and 5: supernatant from transfected CHO-K1 cells; 2: positive control; 3 and 4: mock cell supernatant.

**Figure 4:**
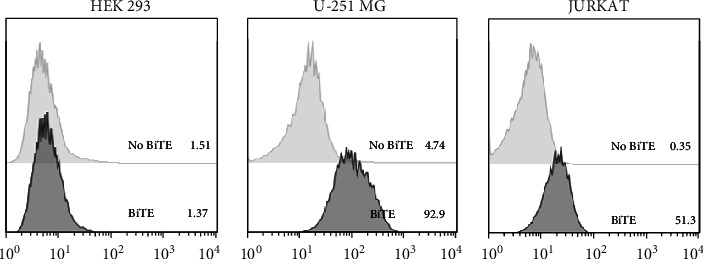
The histogram demonstrated the ability of anti-PDL1-BiTE to directly bind to PDL1on U-251 MG cells (PDL1-positive) and JURKAT cells (CD3-positive) while unbinding to HEK293 cells (as negative cell line).

The anti-PDL1-BiTE was constructed as two single-chain variable fragments (scFv) recombinant protein of the humanized OKT3 anti-CD3 monoclonal antibody and the human avelumab anti-PDL1monoclonal antibody (Figure 2). After anti-PDL1-BiTE gene has been inserted into the pCHO1.0 vector (Supplementary file, Figure 1), the PDL1-BiTE/pCHO.1 construct was transfected into CHO-K1 cells, and stable anti-PDL1-BiTE-producing transfectants were obtained by selection on puromycin. Stable transfectants were grown in serum-free media. Upon expression, these active anti-PDL1-BiTE molecules have one binding site for CD3 and one binding site for PDL1. Native-PAGE showed that fusion proteins were successfully expressed (Figure 3(a)), and a western blot displayed a specific protein band with an approximate molecular weight of anti-PDL1-BiTE (55 kDa) (Figure 3(b)). The amount of anti-PDL1-BiTE released into the culture was measured by ELISA at the indicated time, and the production level was approximately 1.5 *μ*g/mL/8 days. Also, the binding ability of anti-PDL1-BiTE to the PDL1protein in U-251 MG cells, JURKAT cells, and HEK293 cells was evaluated by flow cytometry. First, we confirmed the high PDL1 expression level on U-251 MG cells and low expression on HEK293 cells, which were 93.3% and 1.53%, respectively. Also, expression level of CD3 on JURKAT cells was 60% (Supplementary file, Figure 2). The percentages of anti-PDL1-BiTE binding to CD3 (51.3%) and PDL1pos cell line (92.9%) were much higher than that of the negative cell line (1.37%), indicating that anti-PDL1-BiTE has a high specificity for the CD3 and PDL1protein targets (Figure 4).

### 3.3. Biological Activity of Recombinant Protein Anti-PDL1-BiTE

#### 3.3.1. Anti-PDL1-BiTE Cytotoxic Activity on Tumor Cells

**Figure 5:**
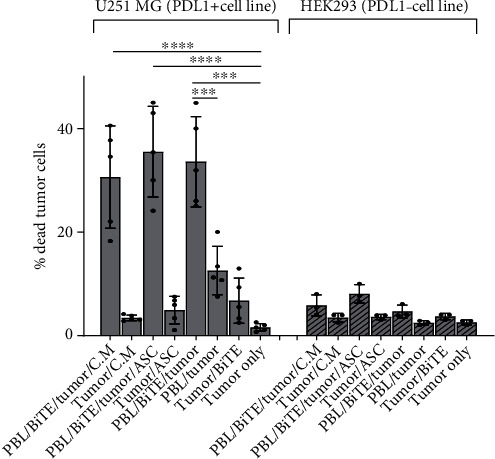
Cytotoxic effect of anti-PDL1-BiTE on PDL1pos and PDL1neg cells. The percentage of dead cells increased in PDL1pos tumor cells especially when BiTE was used. Data was presented as mean ± SD (^∗∗∗^*P* < 0.001 and ^∗∗∗∗^*P* < 0.0001). Data are representative of 5 experiments in U251-MG cell lines and 3 experiments in Hek293 cell lines. ASC: adipose-derived mesenchymal stem cells; C.M.: condition media; PBL: peripheral blood lymphocyte; BiTE: bispecific T cell engager, “anti-PDL1-BiTE”.

Anti-PDL1-BiTE treatment caused a significant increase in T cell killing activity. Approximately 33.5 (±8.7)% of tumor cells were killed in the presence of anti-PDL1-BiTE and PBLs, whereas it was 12.5 (±4.6)% in PBLs/tumor condition (*P* = 0.0001). The killing rate in PBL/BiTE/tumor condition was higher than other conditions that was significant when compared to the tumor only condition (*P* ≤ 0.0001) (Figure 5). Anti-PDL1-BiTE in combination with PBLs, ASCs, and C.M. kills 35.5 (±8.7)% and 30.6 (±9.8)% of PDL1pos tumor cells, respectively. However, this difference was not statistically significant, when compared to PBL/BiTE/tumor (Figure 5). To ensure that the anti-PDL1-BiTE cytotoxic activity is specific to PDL1pos target cells, PDL1-human HEK293 cells were included in the study, and less than 10% of HEK293 cells were killed in different conditions.

#### 3.3.2. Anti-PDL1-BiTE Effects on PBL Proliferation

**Figure 6:**
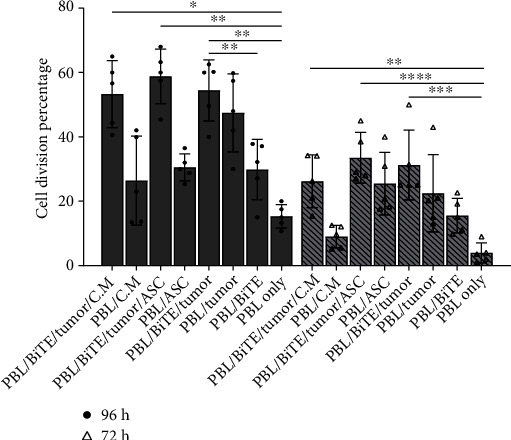
Proliferation of PBLs significantly increased in the presence of anti-PDL1-BiTE/tumor. ASCs and C.M. showed synergistic effects for BiTE. Cell division percentage of PBLs was measured in different conditions, and statistical analysis was performed using a one-way ANOVA test. Data was presented as mean ± SD (^∗^*P* < 0.05, ^∗∗^*P* < 0.01, ^∗∗∗^*P* < 0.001, and ^∗∗∗∗^*P* < 0.0001. Data are representative of 5 experiments. ASC: adipose-derived mesenchymal stem cells, C.M.: conditioned media, PBL: peripheral blood lymphocyte; BiTE: bispecific T cell engager, “anti-PDL1-BiTE”.

We evaluated whether anti-PDL1-BiTE could stimulate PBL proliferation 72 and 96 hours after treatment. According to the findings, the expansion of PBLs began on day 3, when the percentage of cell division was 22.4 (±11.9)% in the presence of tumor and increased to 31.2 (±10.8)% when cocultured with anti-PDL1-BiTE/tumor as compared to 3.9 (±3.0)% in PBLs only (*P* = 0.0002, Figure 6). On day 4, cell division percentage increased to 47.4 (±12.0)% and 54.4 (±9.5)% (*P* = 0.0099), as compared to 29.8 (±9.3)% in PBLs only (*P* = 0.0099, Figure 6). The proliferation of PBLs was in the presence of ASCs and C.M. to 33.5 (±7.9)% and 26.2 (±8.2)% (*P* > 0.05, Figure 6) on day 3 and reached 58.8 (±8.4)% and 53.3 (±10.4)% (*P* > 0.05, Figure 6) on day 4, respectively, as compared to PBL/PDL1-BiTE/tumor alone. However, this difference was not statistically significant (Figure 6).

#### 3.3.3. Anti-PDL1-BiTE and Activation of CD3-Positive Lymphocytes

**Figure 7:**
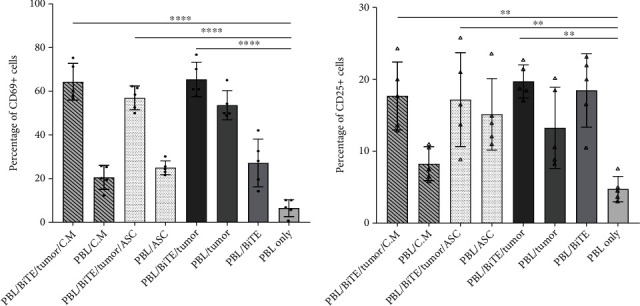
Expression of CD69 (a) and CD25 (b) activation markers. The percentage of CD69+ cells was significantly higher in PBL/BiTE/tumor condition compared to PBL only and PBL/BiTE. The data was presented as mean ± SD (^∗∗^*P* < 0.01 and ^∗∗∗∗^*P* < 0.0001. Data are representative of 5 experiments. ASC: adipose-derived mesenchymal stem cells; C.M.: condition media; PBL: peripheral blood lymphocyte; BiTE: bispecific T cell engager, “anti-PDL1-BiTE”.

Activation markers CD25 and CD69 were upregulated on CD4+ T cells in the presence of PDL1-BiTE/tumor, rather than PBLs/tumor. As shown in Figures 7(a) and 7(a), CD25 and CD69 increased from 13.2 (±5.6)% and 53.6 (±6.6)% in the condition of PBL/tumor to 19.7 (±2.3)% and 65.4 (±7.8)% in the case of PBL/PDL1-BiTE/tumor, respectively. 17.7 (±4.7)% and 17.1 (±6.5)% of the cells showed the expression of CD25 in PBL/PDL1-BiTE/tumor/ASCs or C.M. (*P* > 0.05, Figure 7(b)), whereas these percentages were 56.9 (±5.4)% and 64.3 (±8.4)% for CD69, respectively (*P* > 0.05, Figure 7(a)).

## 4. Discussion

Cancer immunotherapy boosts immune function to eliminate tumor cells and prevent tumor progression. Of all immune cells, T cells are the most powerful cells for directly killing cancer cells [32]. At the present time, various approaches such as bispecific antibodies (bsAbs) have been established to improve T cell responses and their recruitment [39]. Anti − CD3 × anti − CD19 bispecific antibody is one of the promising immunotherapy tools which can efficiently redirect T cells to lyse patient-derived B-ALL cells [40]. BiTEs, as one of the bsAbs and a new strategy for cancer therapy, create a hot TME and enhance antitumor activity [41]. Moreover, it targets other cell types in the TME, such as MDSCs, and provides an additive effect on the entire microenvironment, so it may reduce the dosage or treatment time compared to a single treatment with decreasing drug side effects [32]. In the present study, we demonstrated the feasibility and potential application of the anti-PDL1-BiTE construct derived from a PDL1 mAb (avelumab) and a CD3 mAb (OKT3), for treating PDL1-expressing tumors with a classic structure such as blinatumomab [42]. The hypothesis is that BiTE, as an immunotherapy agent, can recruit T cells to TME and by suppressing PDL1 activates cytotoxic T cells. Additionally, we demonstrated the effect of ASCs and C.M. on BiTE function in a coculture system.

In our study, the binding specificity of both moieties of anti-PDL1-BiTE was confirmed and examined by flow cytometry analysis on PDL1pos/neg cell lines. The anti-PDL1-BiTE binds strongly to the PDL1 antigen in the PDL1pos cell line but not in the PDL1neg cell line. The fact that there was no binding on a negative PDL1cell line confirmed the specificity of anti-PDL1-BiTE binding. In contrast, binding via the anti-CD3 domain to JURKAT cells was less noticeable. The limited binding ability of anti-PDL1-BiTE to the anti-CD3 domain represents a unique feature of the optimized bispecific antibody construct, which is required for repeated retargeting of T cells. Therefore, it is a prerequisite for an optimal killing effect with minimal nonspecific side effects from T cell hyperactivation.

Anti-PDL1-BiTE is a novel reagent with the potential to be a useful therapeutic strategy due to its ability to activate tumor-reactive T cells and direct them to PDL1pos target cells [43]. PDL1 as the ligand of PD-1 is expressed on a wide range of tumor cells, including lung cancer, breast cancer, and glioma [44]. Our results showed that anti-PDL1-BiTE can mediate effector cell attachment to tumor cells, directing polyclonal T cells to kill PDL1pos tumor cells in vitro. The binding of anti-PDL1-BiTE to PDL1pos cells is linearly proportional to the amount of PDL1expressed by the target cells. This result was observed only in U251-MG cells as a PDL1pos cell line which induced T cell cytotoxicity against tumor cells, whereas at equal concentration of anti-PDL1-BiTE and E : T ratio, HEK293 cells as PDL1neg cells had the lowest number of dead cells. These results determine that anti-PDL1-BiTE in combination with PBLs efficiently kills PDL1pos tumor cells while having negligible off-target effects on PDL1neg tumor cells. In correlation with our results, Horn et al. showed that BiTE cytotoxic activity is specific for PDL1pos target cells vs. PDL1neg tumor cells [24]. They used from difference of cell lines PDL1neg human MEL1011 and C8161 cells as PDL1pos, and their results in a cytotoxicity assay showed that less than 11% of MEL1011 cells were killed vs. >50% of PDL1+ C8161 cells. These results demonstrate that anti-PDL1-BiTE is specific for PDL1+ target cells.

The results of Lu et al. showed that MSCs can play an inhibitory role in the vicinity of U251-MG cells [45]. Despite this inhibitory effect of MSCs, we did not observe a significant negative impact of ASCs/C.M. on the performance of anti-PDL1-BiTE. Therefore, it is possible that anti-PDL1-BiTE can overcome the inhibitory nature of ASCs in the TME. Our findings clearly demonstrated an increase in proliferation of PBLs in the presence of anti-PDL1-BiTE and tumor, as evidenced by binding from both sides and restored T cell proliferation which is increased over time till the fourth day. In line with our study, Wathikthinnakon et al. showed an increase in the proliferation of lymphocytes in the presence of anti-PDL1-BiTE, which was amplified with time [43].

In addition, assessment of the expression of the activation markers of lymphocytes showed the higher expression of CD69 in the presence of anti-PDL1-BiTE though we did not observe any obvious difference in the expression of CD25. Regarding CD69, our results were in line with Choi et al.'s study which showed when T cell is incubated with bscEGFRvIII-CD3 and target cells, T cells upregulated surface expression of CD69 [46]. Also, this is in line with the data published by Koristka et al. who showed that after cross-linking via a bispecific Ab, regulatory T cells upregulate the activation markers CD69 and CD25 as well as Treg markers such as CTLA-4 and Foxp3 [47]. Thus, treatment with a single agent may have a negligible suppressive effect on tumors, whereas cotreatment with other immunotherapy methods such as indoleamine 2,3-dioxygenase (IDO) inhibitors or anti-CTLA-4 can have a synergistic effect on cancer treatment and improve patient survival [48, 49].

Most recently, MSCs were discovered to function as immune modulators, which are relatively supposed to be double-edged sword in TME [50]. However, the current study found that anti-PDL1-BiTE causes T cells to proliferate and activation even in combination with ASCs. Contrary to the predictions, even with immune modulatory properties, ASCs could not have a negative effect on anti-PDL1-BiTE performance. Also, C.M. in our evaluation had no noticeable impact on the anti-PDL1-BiTE activity.

The interactions between PDL1pos cancer cells and PD-1pos immune cells, mostly TILs, in tumor environment are complicated [51]. Additionally, the upregulation of immune checkpoints is one of the major mechanisms involved in resistance to BiTE therapy. For instance, TILs dramatically upregulate the expression of PDL1 in melanoma cells by secreting high amounts of cytokines, such as IFN*γ*, which results in the suppression of TILs. In fact, IFN*γ* pathway could be one of feasible pathways, which causes the PDL1 upregulation in cancers [52]. Moreover, the PD-1/PDL1 communication increases IDO in melanoma microenvironment [48] which exhausts T cells of essential tryptophan and suppresses their metabolites, thus leading to CTL inhibition and Treg elevation [53]. Therefore, based on the findings of our study, it is necessary to optimize the BiTE concentration depending on the kind of tumor for a better result. Additionally, as PD-1 and IDO pathway can interfere with PDL1 activity after releasing IFN*γ* in TME [54], this may eventually result in a decrease in the efficacy of immunotherapy. Accordingly, it is suggested that the concentration of BiTE in the environment should be higher than that of PDL1 to overcome its negative effects on the immune system.

In general, it is indicated that utilizing a costimulator, such as CD28/CD80/41BBL in coculture system, might induce BiTE function for proliferation of T cells and induction of CD25/CD69 in lymphocytes [55]. On the other hand, in agreement with our study, Horn et al. [24] and Dreier et al. [56] have claimed that BiTE is an independent costimulator to activate lymphocytes. According to our findings, anti-PDL1-BiTE is already active in the presence of unstimulated T lymphocytes. However, given the absence of lymphocyte stimulators and the state of the tumor, conditioning PBLs with IL-2 and CD28 may further augment the cytotoxic potential of T cells with anti-PDL1-BiTE.

## 5. Conclusion

Altogether, anti-PDL1-BiTE may be promising for the treatment of PDL1-positive tumors due to its capability of boosting T cell accumulation and thus improving the prognosis of PDL1pos cancers. Based on our data, this recombinant protein seems to be promising to overcome the immunomodulatory nature of tumor site caused by different cells including MSCs. As a recombinant protein platform, BiTEs can be industrially produced with current advanced protein production technologies. However, they may need readministration due to BiTEs' short half-life which can be improved using different protein modification strategies.

## Data Availability

All data generated or analyzed during this study are included in this published article.

## Abbreviations

BiTE: Bispecific T cell engager antibodies
VL: Variable light chain
VH: Variable heavy chain
MAb: Monoclonal antibody
MDSCs: Myeloid-derived suppressor cells
PD1: Programmed cell death protein
PD-L1: Programmed cell death ligand
TME: Tumor microenvironment
ECM: Extracellular matrix
TICs: Tumor-initiating cells
CSCs: Cancer stem cells
MSCs: Mesenchymal stem cells
ASCs: Adipose-derived mesenchymal stem cells
PBMCs: Peripheral blood mononuclear cells
HEK293: Human embryonic kidney tumor cell line
FBS: Fetal bovine serum
DMEM: Dulbecco's modified Eagle's medium
PBS: Phosphate-buffered saline
FITC: Fluorescein isothiocyanate
PE: Phycoerythrin
PBLs: Peripheral blood lymphocyte
ASC-C.M: ASC-conditioned medium
Native-PAGE: Native polyacrylamide gel electrophoresis
PVDF: Polyvinylidene difluoride
ECL: Chemiluminescence
ELISA: Enzyme-linked immunosorbent assay
TMB: Tetramethyl benzidine
IDO: Indoleamine 2,3-dioxygenase
CFSE: Carboxyfluorescein succinimidyl ester
7AAD: 7-Aminoactinomycin D
CTLA-4: Cytotoxic T lymphocyte-associated protein 4
41BBL: 4-1BB ligand.

## Supplementary Materials

Supplementary Materials Figure 1: schematic representation of PDL1-BiTE construct inserted into pCHO1 plasmid backbone. Figure 2: U-251 MG and HEK293 cell lines were stained with fluorescent antibodies to PDL1 (29E.2A3 mAb) and Jurkat cells for CD3 or an isotype control mAb and analyzed by flow cytometry. Dark gray shows labeled cells, and light gray shows the isotype or unlabeled.
