# Supplementary material for: Activation of T Lymphocytes with Anti-PDL1-BiTE in the Presence of Adipose-Derived Mesenchymal Stem Cells (ASCs)
Source: Biomed Res Int. 2023 Jun 7;2023:7692726. doi: 10.1155/2023/7692726 (PMC11401667; doi:10.1155/2023/7692726)
Supplement: Supplementary Materials — Figure 1: schematic representation of PDL1-BiTE construct inserted into pCHO1 plasmid backbone. Figure 2: U-251 MG and HEK293 cell lines were stained with fluorescent antibodies to PDL1 (29E.2A3 mAb) and Jurkat cells for CD3 or an isotype control mAb and analyzed by flow cytometry. Dark gray shows labeled cells, and light gray shows the isotype or unlabeled. [file 7692726.f1.doc]

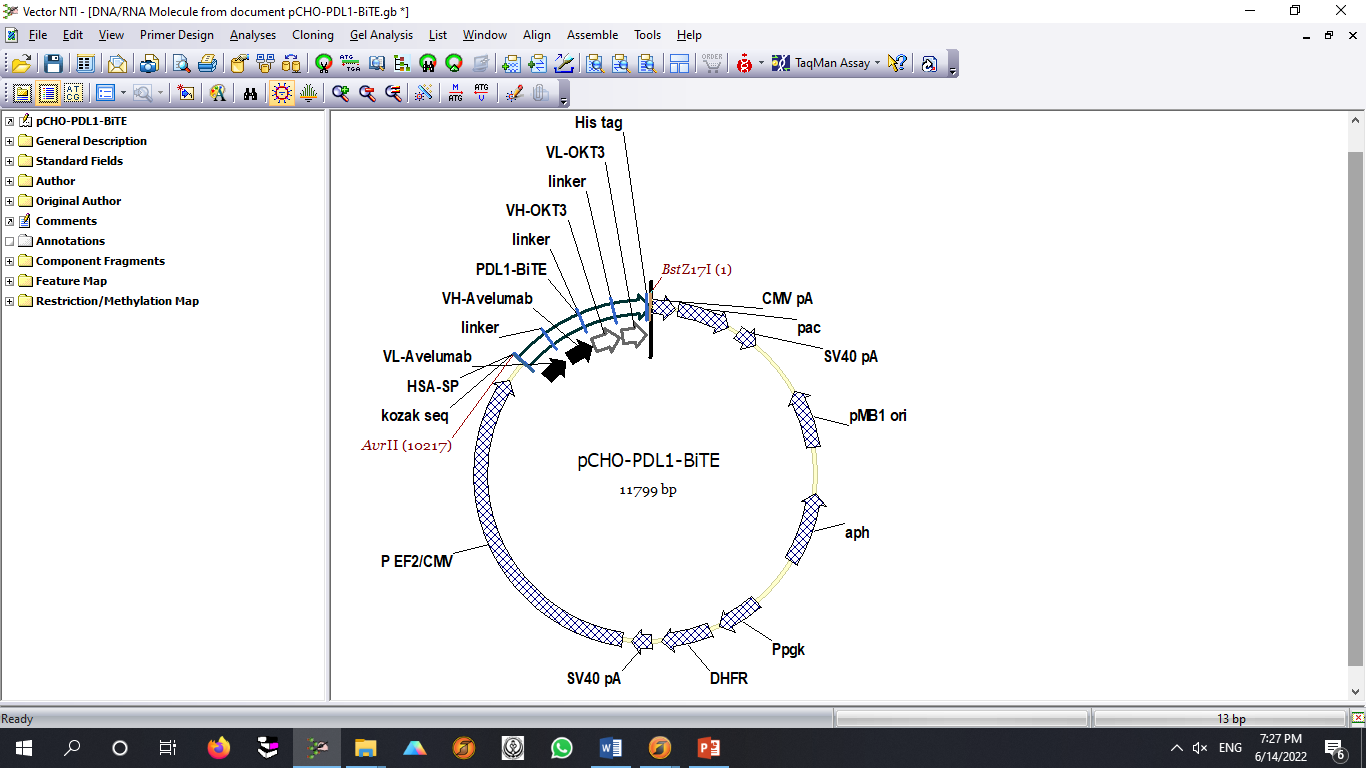


Figure 1: Schematic representation of PDL1-BiTE construct inserted into pCHO1 plasmid backbone.


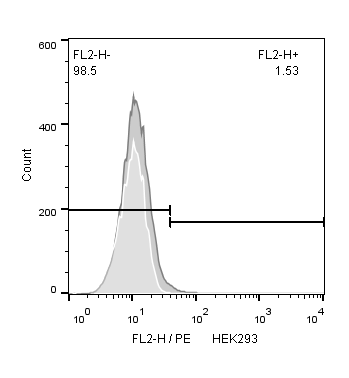

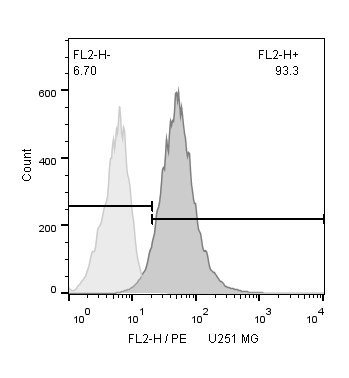

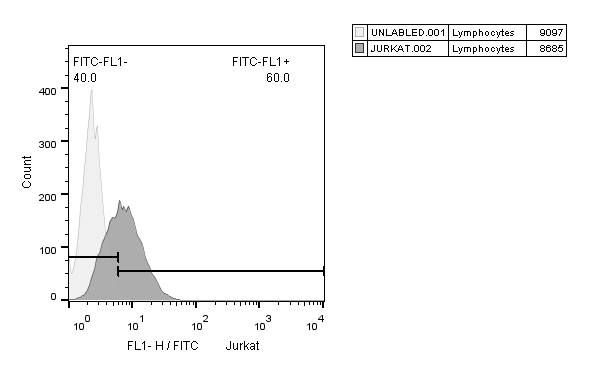


Labeled

unlabeled

Figure 2: U-251 MG and HEK293 cell lines were stained with fluorescent antibodies to PDL1 (29E.2A3 mAb) and Jurkat cells for CD3 or an isotype control mAb and analyzed by flow cytometry. **Dark gray shows** labeled cells and light gray shows the isotype or Unlabeled.
